# Research on the Analysis and Application of Polymer Materials in Contemporary Sculpture Art Creation

**DOI:** 10.3390/polym15122727

**Published:** 2023-06-18

**Authors:** Chao Gao, Feng Wang, Xiaobing Hu, Ming Zhang

**Affiliations:** 1 School of Design, Jiangnan University, Wuxi 214000, China; 2 Fine Art School, Anqing Normal University, Anqing 246001, China; 3 School of Design and Art, Jingdezhen Ceramic University, Jingdezhen 333403, China

**Keywords:** polymer material, contemporary sculpture, art creation, creative material

## Abstract

The application of polymer materials in sculpture art creation is extensive and plays a significant role in the development of sculpture art. This article aims to systematically explore the application of polymer materials in contemporary sculpture art creation. The research comprehensively applies various techniques such as literature research, data comparison, and case analysis to explore in detail the ways, methods, and paths of polymer materials employed in the shaping, decoration, and protection of sculptural artworks. First, the article analyzes three methods of shaping sculpture artworks with polymer materials (casting, printing, and constructing). Secondly, it explores two techniques of using polymer materials to adorn sculpture artworks (coloring and imitating texture); then it discusses the significant approach of using polymer materials to protect sculptural artworks (protective spray film). Finally, the research identifies the merits and demerits of using polymer materials in contemporary sculpture art creation. The findings of this study are expected to enrich the effective application of polymer materials in contemporary sculpture art creation and offer novel techniques and ideas for contemporary sculpture art creators.

## 1. Introduction

Polymer materials are a class of materials that consist primarily of macromolecular compounds. These compounds include plastics, rubber, fibers, coatings, adhesives, and polymer-based composites [1]. According to their sources, polymeric materials can be divided into natural and synthetic polymeric materials [2,3]. Natural polymer materials are derived from natural sources such as natural fibers, plant gums, and natural resins [4], synthetic polymer materials are manufactured and include plastics, synthetic rubber, and synthetic fibers [5]. Natural polymer materials like wool, linen, cotton, hair, and silk have been used as raw materials in human creative activities for centuries [6]. Since the 20th century, synthetic polymeric materials such as unsaturated polyester resin, acrylic ester, epoxy resin, polyurethane, polyethylene, polypropylene, and organosilicon have gradually become the main materials for modern industrial large-scale production [7]. With the development of technology, synthetic polymer materials have been widely used in various fields, including but not limited to automobiles, ships, the military, aerospace technology, product manufacturing, biomedicine, architectural space, sports equipment, etc. [8,9]. Currently, polymer materials have permeated every aspect of human life and every corner of the world, becoming an important element supporting the development of human society [10].

Polymer materials have gained significant popularity in contemporary art, being utilized in diverse fields such as painting [11], public art [3], and ceramic art [12]. The use of polymer materials in the creation of contemporary sculptures is also prevalent, leading to the production of numerous exceptional works that hold great importance for the advancement of contemporary sculpture art.

The art of sculpture is a static three-dimensional art form that is realized through the use of material substances. The image of a sculpture is realized through the use of material substances, which impart to it a unique texture and beauty [13,14]. As such, materials play a pivotal role in the creative process of sculpture and serve as the foundation for sculptors in creating their works. Polymer materials, which possess superior properties in plasticity, impact resistance, weather resistance, and corrosion resistance, as well as being less expensive than traditional materials, have become increasingly popular among contemporary sculptors. However, despite their growing popularity, contemporary sculptors may possess limited knowledge regarding polymer materials and their systematic application in sculpture creation, which may hinder their artistic output. Therefore, it is vital to study the application of polymer materials in contemporary sculpture art.

Despite scholars examining the application methods [15] and processes [16] of polymer materials in contemporary sculpture art from various perspectives, as well as investigating the composition and manufacturing technology [17] of polymer materials in contemporary sculpture and their impact on the sculpture art form [18], a comprehensive study regarding the application of polymer materials in contemporary sculpture creation has yet to be conducted. As materials science advances and contemporary sculpture art develops, the application of polymer materials in this field has become increasingly frequent, thus emphasizing the need for a systematic exploration of how polymer materials can be applied in the creation of contemporary sculpture art. The objective of this article is to conduct a comprehensive analysis and discussion of the various ways, methodologies, and approaches employed in the use of polymer materials in contemporary sculpture art from an interdisciplinary perspective. The article employs a comprehensive approach that involves literature research, data comparison, and case analysis in order to thoroughly examine the various ways in which polymer materials may be utilized in the creation, decoration, and protection of sculptures. Furthermore, the article will assess the scientific value and limitations of polymer materials in contemporary sculpture art creation. The findings of this research will provide useful insights and guidance for the effective use of polymer materials in contemporary sculpture art creation and offer valuable means and ideas for contemporary sculpture art creators.

## 2. Polymer Materials and Their Applications in Creating Sculpture Artistic Creation

In contemporary sculpture art, polymer materials serve as raw materials for making sculpture artworks. The selection of appropriate materials is the foundation of artistic creation, as any artwork that fails to be conveyed through its materials remains a mere figment of imagination [3,19]. The process of creating sculptural works begins with the careful selection of materials, through which artists can realize their creative concepts and plans. Polymer materials have expanded the range of raw materials available for use in sculpture art, rendering the creative methods and means of this art form more diverse. In particular, there are three primary ways in which polymer materials can be employed as raw materials for creating sculpture art: mold casting, 3D printing, and construction molding. As such, polymer materials can function as mold casting materials, 3D printing materials, or construction molding materials, all of which contribute to the modeling of sculpture artworks. This prompts the question of which specific polymer materials are suitable for use as mold casting materials, printing materials, and construction materials in the creation of sculpture artworks and how they can be effectively applied.

### 2.1. Polymer Materials and Their Applications as Mold Casting Materials for Sculptural Artworks

Mold casting is a prevalent technique in the creation of sculptures. In traditional sculpting, artists employ malleable materials such as clay to produce a model of the sculpture to fully realize their creative ideas and concepts. Subsequently, a range of materials are used to produce the sculpture model, which is then refined to create the final sculpture. In modern sculptural art, synthetic polymer materials are frequently utilized as the reproduction material for sculptures. Polymers with elasticity, such as resin, plastic, and fiber, can all be used to create the sculpture model. Currently, the most commonly employed casting materials for sculptures in contemporary sculptural art is glass fiber-reinforced plastic (GFRP), which is made from various synthetic polymer materials.

GFRP is a composite material composed of unsaturated polyester, epoxy resin, phenolic resin, and other thermosetting or thermoplastic resins that are strengthened with glass fibers [20]. This material is renowned for its light weight, high strength, corrosion resistance, and ease of processing. GFRP sculptures exhibit exceptional durability, aesthetic appeal, vivid hues, ease of production and installation, and the ability to simulate various material effects to meet the demands of different designs and themes. In comparison to traditional materials, GFRP boasts outstanding advantages [21,22]. GFRP displays excellent resistance to atmosphere, water, and typical concentrations of acid, alkalis, salt, and oil substances. These properties make it a suitable material for the long-term exhibition and preservation of sculptures [23]. By comparing the basic physical performance data of GFRP with commonly used metal materials such as copper, stainless steel, and iron for sculpture creation (see Table 1), we find that: firstly, GFRP material has strength, stability, and compression resistance that are not less than metal materials, despite weighing only about 1/4 what metal materials do [20]. Secondly, under the same environmental conditions, GFRP exhibits lower thermal conductivity and superior thermal performance, making it an effective insulation material [23,24]. Thirdly, it is highly malleable, allowing for various shapes to be formed with ease [25]. Lastly, GFRP possesses strong heat and corrosion resistance, as well as chemical stability in various acidic and alkaline environments [25]. Related research found that GFRP can maintain its strength even at temperatures as high as 300 °C and can last up to 20 years in harsh environments such as high temperature, strong light, and heavy rain [26].

In the manufacture of fiberglass, glass fibers are employed as the reinforcing material while the matrix material is made up of thermosetting resins, including unsaturated and epoxy resins. Talcum powder is also incorporated as filler material, and polyamide resins are utilized as curing agents to solidify the matrix material. During production, the unsaturated and epoxy resins, which function as matrix materials, serve as adhesives to bond the talcum powder and glass fibers with the matrix material. Subsequently, polyamide resins, acting as curing agents, rapidly solidify the matrix material, resulting in a lightweight cured polymer structural material [31]. Notably, the strength, corrosion resistance, and heat resistance of GFRP material are determined by the glass fibers [25,32]. It is a type of inorganic fibrous material that is derived from molten glass through either drawing or blowing processes. These processes result in the formation of long filaments, short filaments, or flocculent shapes with a diameter that typically ranges from 3 μm to 80 μm. The primary chemical components of glass fiber include silica, alumina, boron oxide, magnesium oxide, and sodium oxide. Glass fiber is known for its impressive strength, with a tensile strength as high as 3600 Mpa for fiber with a diameter of 10 μm. This strength is equivalent to being able to withstand a tensile force of 360 kg/mm^2^, which surpasses that of steel [20,33]. The excellent properties of fiberglass are precisely what give GFRP its desirable characteristics, such as a higher elasticity modulus and greater ease of processing. The performance of the resin also plays a crucial role in determining the artistic effect and service life of GFRP. Among the GFRP materials used for sculpture art creation, the most commonly used resin materials are 191# unsaturated polyester resin, 196# unsaturated polyester resin, and epoxy resin [16,18]. A comparison of the fundamental properties of these resins, such as viscosity, shrinkage rate, and acid value, as presented in Table 2, shows significant variations that result in distinct properties of the final fiberglass materials. Talc is a frequently used filler material in the production of high-density polymer materials. It comprises hydrated magnesium silicate with a chemical formula of Mg_3_[Si_4_O_10_](OH)_2_ and boasts exceptional physical and chemical characteristics, including lubricity, fire resistance, acid resistance, insulation, high melting point, low chemical reactivity, good covering power, softness, good gloss, and strong adsorption ability [34,35]. During the process of fabricating fiberglass using resin, the curing agent is an indispensable component [36], and the combination of the resin material with the curing agent is critical to the production of a sturdy material [37]. Polyamide resins are an excellent choice for resin curing agents [38]. In GFRP preparation, talc, which functions as a filler, is typically combined with the resin at a ratio of 1:1 [16].

In brief, GFRP exhibits physical characteristics that are comparable to those of metallic materials, yet is cost-effective and malleable. It is a lightweight material that is easily transportable and possesses robust corrosion resistance. Furthermore, it overcomes the shortcoming of plastic softening when subjected to high temperatures and can better achieve the aesthetic effect of sculptural works. These attributes render it a frequently utilized material for replicating artworks in modern sculptural art, particularly for sculptures situated indoors or temporarily placed outdoors.

### 2.2. Polymeric Materials and Their Applications as 3D Printing Materials for Sculpture Artworks 

In recent years, the development of material science and digital technology has led to a new form of sculptural art creation—the production of sculpture by printing. Currently, the primary method of 3D printing employed in the production of sculptural works is a novel form of additive manufacturing technology which utilizes materials such as wire–plastic or powdered metal that can be selectively bonded and accumulated layer by layer to form a solid object. This technology has found wide application in diverse fields such as architecture, medicine, and industry [42]. 3D printing technology has various types, mainly fused deposition modeling (FDM), light-cured stereolithography (SLA), selective laser sintering (SLS), and 3D inkjet printing (3DP) [43,44]. As 3D printing technology matures, more and more artists are opting to utilize this method to process and produce their own sculptural works of art.

When using 3D printing technology to create sculptural artworks, the selection of 3D printing materials is of utmost importance. Currently, the materials available for 3D printing sculptural art are primarily composed of metal and polymer materials. In comparison, metal materials present costly printing expenses and require higher technical specifications, while polymer materials boast comparatively cheaper printing costs and lower requirements for printing technology and conditions. Moreover, creators can customize polymer materials’ colors according to their needs, thereby presenting better visual effects of their printed sculptural art. Therefore, polymer materials are typically the preferred choice for contemporary artists in the use of 3D printing technology for the creation of sculptural art.

Many scholars have extensively studied the physical properties of polymer materials commonly used in 3D printing [45,46]. At present, the most commonly used polymer materials for 3D printing sculptures include thermoplastic polymer filament materials, photosensitive resins (UV resins), and polymer powders [42,43]. The thermoplastic polymer filament materials mainly comprise acrylonitrile butadiene styrene copolymer (ABS), polylactic acid (PLA), polyvinyl alcohol (PVA), and polycarbonate (PC). The polymer powders can be further classified into thermoplastic polymer powder, thermosetting polymer powder, and polymer composite material powder [42,43,47]. Each of these polymer materials has distinct characteristics, suitable 3D printing technologies, and molding effects, which are shown in Table 3. Therefore, creators should choose the appropriate polymer materials based on their specific creative needs.

The aforementioned high polymer materials, which are frequently employed in 3D printing, possess certain limitations. For instance, ABS, despite its chemical stability, may undergo curling upon surpassing a surface temperature of 240 °C, ultimately impeding the accuracy of the 3D printing process [47]. PLA, on the other hand, is less prone to curling, yet exhibits lower strength, inferior toughness, and reduced crystallinity, all of which can contribute to a negative impact on the printing outcome. [48]. Although PVA offers numerous benefits in film-forming and environmental protection, its main polymeric chain contains many lateral hydroxyl groups that create multiple hydrogen bonds. This results in a melting temperature of approximately 230 °C, close to the decomposition temperature (230–250 °C). As a result, PVA is difficult to process through melting [49,50]. On the other hand, PC has the advantages of ABS, as well as good flame retardancy, low shrinkage, and high mechanical strength [51], and does not emit toxic or unpleasant gases during printing [47]. However, the high number of benzene ring structures in the PC molecule chain prevent chain movement and result in high melt viscosity. This leads to disadvantages such as poor toughness, poor compressive strength, and low printing accuracy [52,53,54]. Photosensitive resin is the most commonly used material in SLA. However, due to the fact that most photosensitive materials are initiated by free radicals, the resultant cured photosensitive resin exhibits properties of brittleness and hardness and is limited in its application due to its poor bending and stretching capabilities [47]. Polymer powders such as high-performance thermoplastic powder, thermosetting polymer powder, and polymer composite powder are primarily subject to the constraints and limitations inherent in SLS technology [42].

In the field of materials science, there have been several studies conducted by many researchers to modify relevant polymer materials used in 3D printing. These modifications have addressed the shortcomings of the materials and have subsequently improved the molding quality and efficiency of 3D printing. For example, Zhou Ming’an et al. produced ABS/nano-TiO_2_ by utilizing ABS as the matrix and nano-TiO_2_ as the modifier. Tests have indicated that the modified composite material displays superior mechanical properties, and all properties remain stable after 3D printing [47,55]. Similarly, Qiu Jun et al. developed a polymer composite material by blending PC with ABS, which significantly reduces the material shrinkage rate, enhances material adhesion, and greatly improves the quality of 3D printed products while also reducing costs [43]. The combined properties of PC and ABS mixed materials, such as impact resistance and processability, make them an ideal material for 3D printing [56]. Junyang S et al. have produced a photosensitive resin with high tensile strength by employing epoxy resin, acrylic resin, and photoinitiators of free radical and cationic nature [42]. Yang Guisheng et al. have also generated a polymer material that exhibits improved bending strength and reduced shrinkage by modifying photosensitive resin with nylon microspheres. As a result, the modified materials facilitate faster molding speed, higher mechanical strength, and better dimensional stability of 3D printed products [57]. These materials have been applied to contemporary sculpture art creation through 3D printing technology, thereby enhancing the quality of sculpture works. Furthermore, the features of these modified materials enable contemporary artists to employ 3D printing to manufacture sculptures with more intricate structures.

### 2.3. Polymer Materials and Their Applications as Construction Materials for Sculpture Artworks

Sculptural works of art made by molding have become a popular technique among contemporary sculptors. The use of polymer materials to construct sculpture artworks has become a pivotal choice for many sculptors. Polymer materials offer a diverse range of types and colors, as well as a high level of plasticity, corrosion resistance, ease of processing, and a modern aesthetic. These advantages render polymer materials the most frequently employed construction material in the creation of sculptural art by many contemporary sculptors.

When producing sculptures through the process of constructing and shaping, artists can choose materials according to their creative concepts and plans and then effectively blend said materials in a skillful manner to yield a finished piece. In contemporary sculpture art, both naturally occurring and synthetic high molecular weight materials are frequently utilized. Regardless of their origin, these materials serve as a powerful medium through which sculptors are able to convey their imaginative concepts and ideas. 

**Figure 1:**
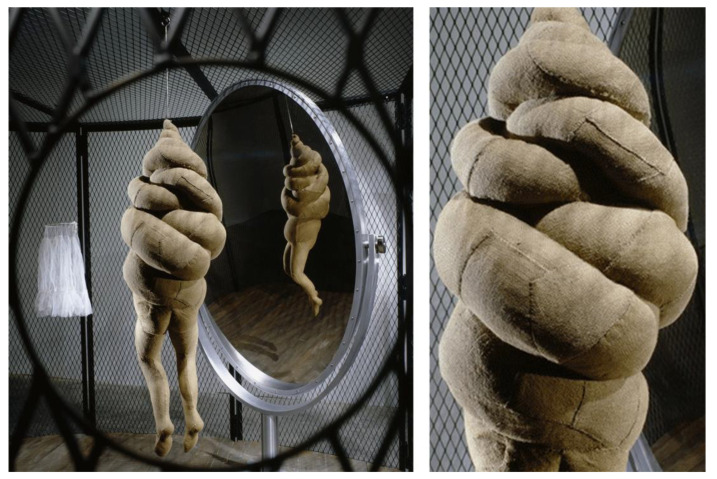
Louise Bourgeois/*Cell XXVI*/Fabric.

**Figure 2:**
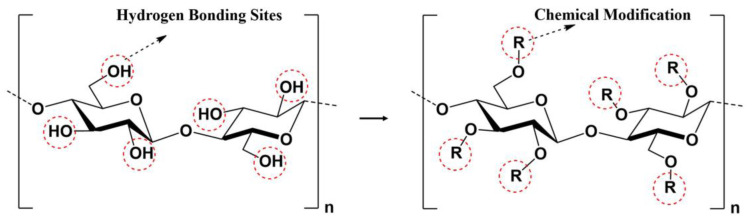
The chemical structure of cellulose and its chemical modification structure.

For example, the sculptural work of art *Cell XXVI* by American artist Louise Bourgeois (see Figure 1) is constructed using a natural polymer material—hemp fiber. The main chemical component of hemp fiber is cellulose, with a molecular formula of (C_6_H_10_O_5_)_n_, which is composed of multiple glucose units [58]. Cellulose, the most abundant biopolymer material on Earth, possesses unique multidimensional physical structures and controllable surface chemistry advantages. It is widely distributed in biomass resources such as green plants. The hydroxyl functional groups on cellulose’s surface facilitate the formation of intermolecular hydrogen bonds, which promotes the ordered arrangement of molecular structures and enables cellulose to exhibit strong mechanical properties. Cellulose also displays high stability under extreme environmental conditions, including high temperature differences, high humidity, high radiation, high corrosion, and high impacts. Furthermore, various chemical modifications can be carried out using the hydroxyl functional groups on cellulose’s surface, which allows for the preparation of materials with various processing properties. Figure 2 shows the chemical structure of cellulose and its chemical modification structure. The chemical and physical properties of cellulose make hemp fiber an excellent material for artistic creation. In contemporary sculpture, artists employ hemp fiber not only as the primary material for shaping their works, but also as a form of communication. In the case of Louise Bourgeois’ sculpture *Cell XXVI*, hemp fiber serves both as the raw material for the artwork and as the language through which the artist conveys her ideas. Bourgeois deliberately chose soft materials such as clothing to create her sculpture, with the aim of dissolving the sense of separation and abandonment that had plagued her throughout her life, and, in so doing, come to terms with her past.

**Figure 3:**
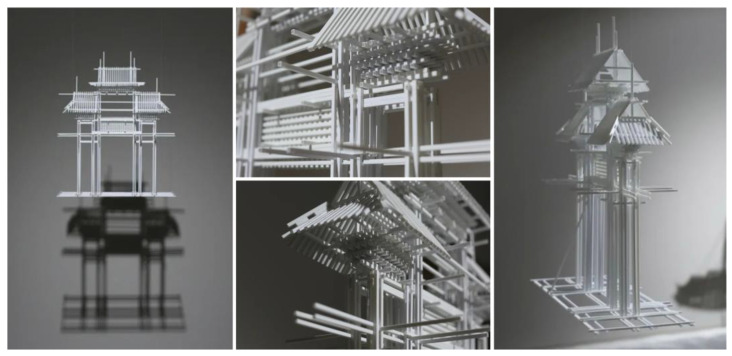
Dong Shubing/*Gou*/PVC pipe.

In contrast, the sculpture *Gou* (Figure 3) by the contemporary Chinese sculptor Dong Shubing is constructed using synthetic polymer material, specifically PVC plastic pipes. PVC is a thermoplastic resin material whose main component is polyvinyl chloride. It is produced by the polymerization of vinyl chloride monomer (VCM). Polyvinyl chloride (PVC), a synthetic material that is currently popularly utilized in various applications such as pipes, film, soft goods, packaging, and others, displays exceptional performance in terms of mechanical properties, acid and alkali resistance, and chemical stability. In this work, the synthetic polymer material, specifically PVC plastic pipe, is not only employed as the raw material for sculptural works, but also functions as a mode of expression for the author to convey their own thoughts and ideas. The artist utilized the archway, a traditional Chinese architectural element, as the foundation for the design of their sculpture. By splicing together pieces of PVC pipes, a polymer building material commonly used in the modern market, the artist was able to combine this material with the ancient Chinese architectural style of the archway. The use of PVC pipes, with their unique texture and color, helped to create a contrast with the archway and enabled the artist to fully express their thoughts and ideas through the artwork. The material texture and color of the PVC pipes themselves weakened the realistic portrayal of the archway, allowing the artwork’s intended concepts and cultural implications to be effectively displayed. The linear arrangement and combination of the PVC pipes created a dynamic visual effect and presented a subtle, yet elusive, visual experience. 

In the process of sculpting, polymer materials serve not only as raw materials but also as adhesives for combining different materials. Polymer adhesives are commonly used in sculpting techniques such as molding and printing. Historically, natural polymers such as peach gum, pine gum, insect gum, and fish gelatin have been used as adhesives for creative activities such as architecture. As materials science has progressed, many synthetic polymer-based adhesives have been extensively used in creative activities due to their superior performance, stronger adhesion, ease of use, and reasonable cost. Among these, epoxy resin AB glue and EVA resin hot melt glue are the most commonly used polymer adhesives in contemporary art creation [59,60,61]. These two types of polymer adhesive differ in their preparation methods, material characteristics, and scope of application, as shown in Table 4. In contemporary sculpture art creation, both types of polymer adhesive are widely used, particularly when using synthetic polymer materials as the basic raw materials to create sculpture artworks. Adhesives made from synthetic polymer materials play a crucial role in this creative process.

## 3. Polymer Materials and Their Applications in Decorating Sculptural Artworks

In contemporary sculpture art, artists often utilize polymer materials to decorate their sculptures. While sculpture art is a three-dimensional art form, decoration plays a significant role in perfecting contemporary sculpture art. Coloring is the main approach to decorating sculpture works.

In the long history of sculpture, color has always played a pivotal role. Sculptors and craftsmen meticulously handle the sculpture’s form and space, as well as pay close attention to coloring their works. In the creation of sculpture art, color serves two purposes: one is to realistically depict the physical properties of objects, and the other is to express the sculptor’s subjective emotions or personal feelings, or as a carrier with certain symbolic significance. [62] Color decoration has the potential to enhance the visual properties and recognition of sculpture works, bringing a strong visual experience. By altering colors, it is possible to imitate other materials, alter the texture of the sculpture material itself, and thereby improve the artistic effect of the sculpture’s shape and surface [63].

Polymer materials, especially synthetic polymer materials, have become the preferred materials for color decoration of sculptures by sculptors due to their vibrant hues, robust adhesion, and user-friendly nature. Contemporary sculptors commonly use acrylic pigments and spray-paint pigments as their go-to polymer coloring materials. These agents can be applied to almost all materials commonly used in sculpture making, such as wood, metal, and fiberglass.

**Figure 4:**
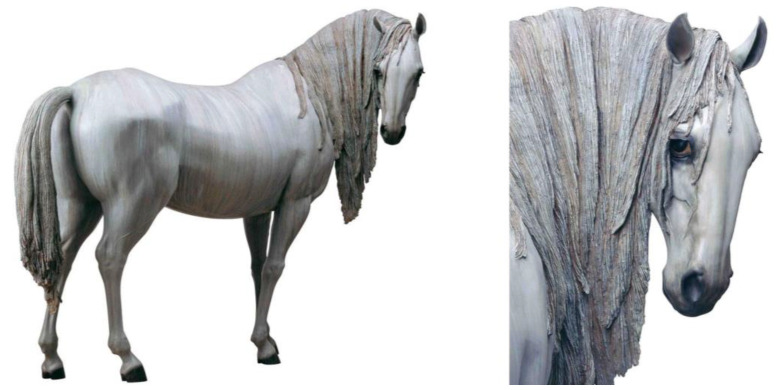
Xiang Jing/*The Otherworld—Will Things Ever Get Better*/GFRP.

Acrylic paint is a dispersed painting pigment composed of poly (methyl methacrylate) that can be dissolved in mineral spirits. It is a multicomponent composite system that utilizes acrylic resin emulsion as a binder and inorganic or organic pigments as coloring agents. The particle size of the solid particles in this multicomponent composite system of acrylic paint is typically controlled to be around 100–200 nanometers. By incorporating surfactants, stable emulsion systems with smaller particle sizes can be formed, effectively preventing particle settling and ensuring long-term pigment stability. Acrylic paint offers several benefits, such as being dilutable with water, quick-drying, long-lasting, vivid in color, easy to blend, rich in color layers, able to depict details, easy to clean, and insoluble in water after drying. Therefore, many sculptors prefer to use acrylic paint to color their works of art, as it allows them to express the delicate texture and rich layers of color in their sculptures. One notable example is the work of the renowned Chinese sculptor Xiang Jing, who hand-paints the majority of her sculptures using acrylic paint, layer by layer, to create a refined sense of depth and a vividly realistic visual effect [64]. In Xiang Jing’s sculpture series known as *Otherworld*, the utilization of acrylic paint has resulted in a visually striking display. In particular, the piece entitled *The Otherworld—Will Things Ever Get Better* (see Figure 4) has been adorned with acrylic paint in a meticulous manner, enhancing the realism of the work and accentuating its artistic merit. 

**Figure 5:**
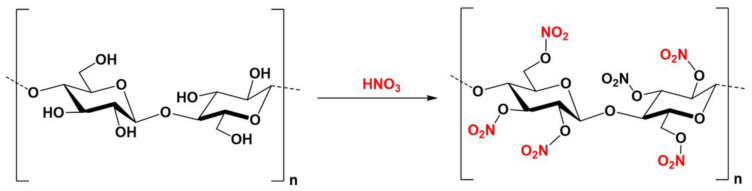
Formation of cellulose nitrate esters via reaction between free hydroxyl groups and nitric acid in cellulose.

Spray-paint pigment is an artificial paint made of high-polymer materials such as nitrocellulose, resins, pigments, and solvents. Its primary film-forming substance, nitrocellulose, is obtained by chemical nitrification of natural cellulose. During this process, the free hydroxyl groups in cellulose react with nitric acid to form cellulose nitrate (as shown in Figure 5). Nitrocellulose that has undergone nitration can be dissolved in low boiling point volatile solvents such as acetone and ether to form a gel-like liquid. When the solvent evaporates, the remaining solute forms a uniform film. These characteristics of nitrocellulose make it a suitable material for use in spray paint pigments.

**Figure 6:**
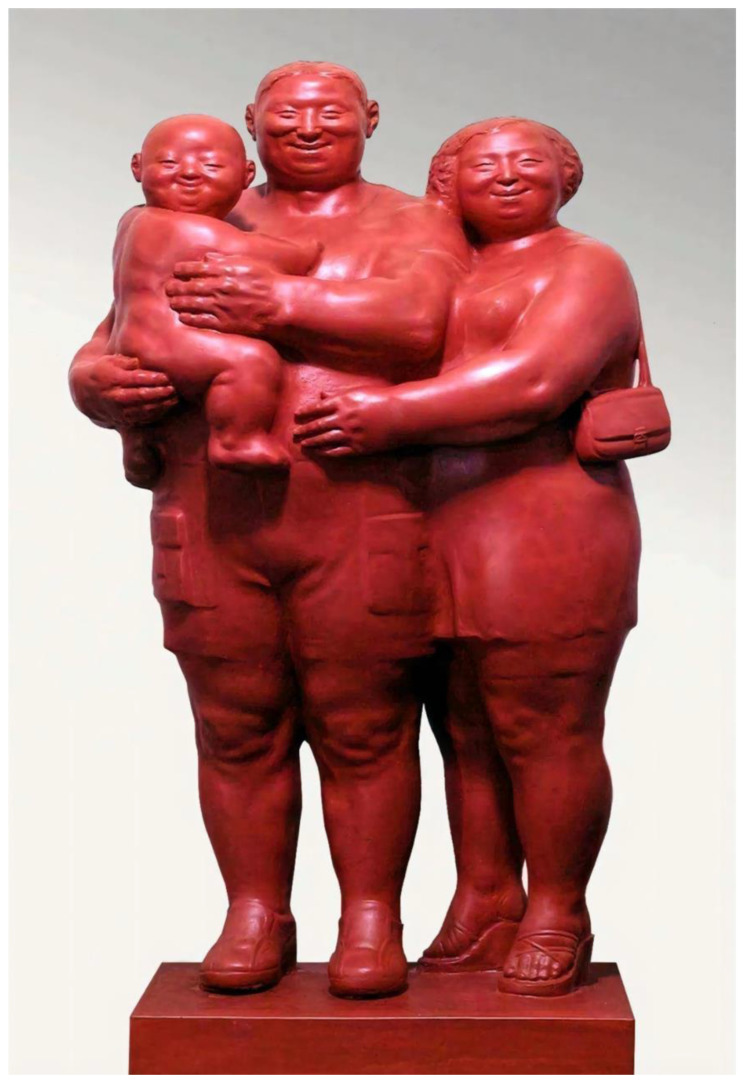
Zhou Acheng/*Afu Family’s Travel 1*/FRP.

This type of pigment is applied to the surface of an object by dispersing the paint into uniform and fine mists using a spray gun or disc atomizer with the help of pressure or centrifugal force. Spray-paint pigments offer a number of advantages, including good fullness, rapid drying, high gloss, strong hardness, favorable film-forming properties, robust adhesion, high spray rate, optimal atomization effects, uniform coloring, and convenient application. This makes them a popular choice for sculptors seeking a uniform, single-color finish for their artworks. For example, the famous Chinese sculptor Zhou Acheng’s sculpture *Afu Family’s Travel 1* (see Figure 6) is decorated with red spray-paint. In Chinese culture, red symbolizes celebration and positivity, while Afu’s family is a traditional and classic Chinese character representing happiness and beauty. The solid red color adhering to the sculpture’s form enhances the accuracy, rationality, originality, and artistic effects of the work’s theme, ideas, and concepts [65]. Currently, automatic spray paint that seals paint and gas in a can is widely available on the market and easy to purchase. It is very convenient to use by pressing the automatic spraying device for spraying.

**Figure 7:**
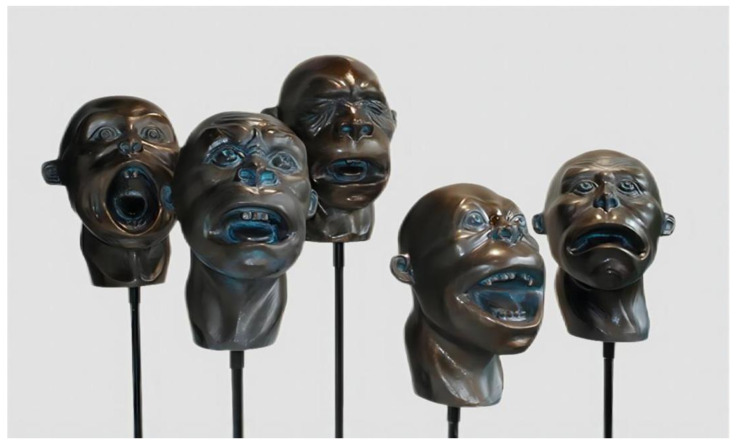
Gao Chao/*Hi! Buddy*/FRP.

Undoubtedly, different polymer materials have their own shortcomings and limitations. Combining different polymer materials can effectively avoid the limitations of individual materials and enable their advantages to be fully realized. For example, acrylic paint and spray paint, while having several advantages for coloring and embellishing sculptures, are also associated with certain limitations. Acrylic paint may present challenges in achieving uniform coloration when applied thinly and may develop cracks if applied too thickly. On the other hand, spray paint may not be ideal for detailed work and may exhibit limited color layer variations. Therefore, many sculpture artists will combine the two in their sculptural art creations. For instance, in the creation of the sculpture art series *Hi! Buddy* (see Figure 7), the artist uses a combination of spray paint and acrylic paint for coloring. First, the artist applied a basic color to the sculpture’s surface via spray-paint and subsequently enhanced it with intricate details using acrylic paint. This method yields a sculpture artwork made of fiberglass, which showcased a texture akin to bronze.

Overall, in contemporary sculptural art, pigments and dyes are the most commonly used polymer coloring materials. Along with acrylic and spray paint, fluorocarbon paint is commonly employed to color stainless steel, while colorants are used to dye resin materials. Due to the wide array of polymer pigments and dyes available, variations in material properties and coloring techniques exist, requiring artists to carefully select the appropriate polymer coloring materials based on their individual creative concepts and plans.

In addition, in modern sculpture art, replicating the texture of various materials with the use of polymer materials is a significant technique for enhancing the visual appeal of sculpture works. The surface texture of the material can elicit physiological responses in individuals through their perceptual system, providing them with relevant information about the material’s features [66]. Therefore, contemporary sculptors attach great importance to the selection of sculpture materials and the processing of surface texture in their works. The wide range of polymer materials available, along with their diverse colors, can be utilized to create various composite materials and achieve a range of texture effects, allowing for the replication of the texture of different materials. This feature of polymer materials can be employed in sculpture creation to give materials such as fiberglass the appearance of wood, bronze, or stone. Therefore, the technique of imitating the texture of different materials using polymer materials is highly valued by contemporary sculptors as an effective decorative method.

## 4. Polymer Materials and Their Applications in Protecting Sculptural Artworks

After the completion of contemporary sculpture, artists usually have to consider the protection of their artwork. This holds especially true for sculptures situated in public spaces, such as landscape sculptures, which endure prolonged exposure to natural and artificial elements including light, rain, and temperature fluctuations, rendering them vulnerable to corrosion, oxidation, and damage. As such, specialized protective measures must be taken. One example is stainless steel sculptures commonly found in outdoor public spaces. Stainless steel sculptures that are often seen in public outdoor spaces have a chromium passivation film on their surface that makes them resistant to rust. However, localized corrosion can occur when acidic, alkaline, or salty substances adhere to the surface of these sculptures. In addition, if metallic element-containing dust settles on the surface of stainless steel, it can cause electrochemical reactions in humid air, damaging the protective film and leading to corrosion. Similarly, when the air is contaminated with sulfides and carbon oxides, these substances form acidic liquids when combined with water vapor in the atmosphere, leading to chemical corrosion of stainless steel. As a result, protective treatment is essential for stainless steel sculptures and even more so for sculptures made of other materials.

Polymer materials, with their remarkable corrosion resistance, oxidation resistance and compressive strength, are considered ideal for safeguarding sculpture works. In contemporary sculpture creation, protective films made of high-polymer materials are commonly used for preserving the art pieces. There are three main ways to spray polymer materials on the surface of sculptures to form a protective film: (1) air spraying method; (2) thermal spraying method; (3) high-pressure airless spraying method. The air spraying method is to spray the polymer coating directly on the surface of the object with the help of negatively compressed air from an air compressor. Thermal spraying is similar to air spraying, except that the polymer coating and the compressed air are preheated before spraying. High-pressure non-air spraying works by compressing the high-polymer coating with a high-pressure pump and then using a specially designed nozzle to apply the coating with a strong impact force to the object surface. All three methods are used in contemporary sculpture art creation, with air spraying and thermal spraying method being the most widely used and the thermal spraying method achieving the best results. Regardless of the technique employed for spraying high polymer materials, the surface of the sculpture works must be clean and smooth before application to ensure effective protection.

**Figure 8:**
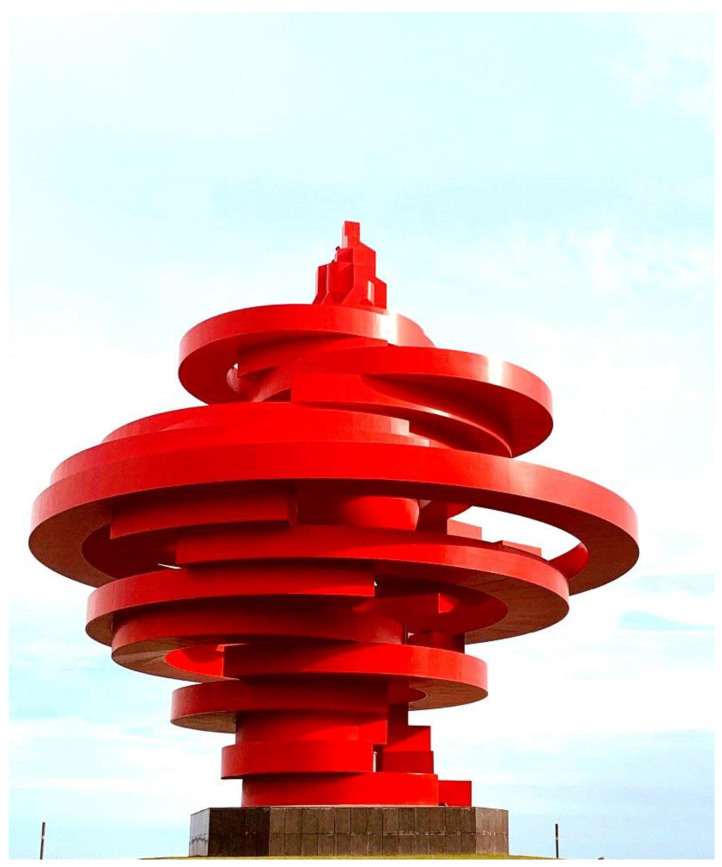
Huang Zhen/*The Wind of May*/Steel.

Due to the fact that different sculpture works made of different materials require different types of protection, the selection of polymer materials used by creators will also vary. In the case of sculptures made of metal materials, which exhibit high strength but are susceptible to weather damage and corrosion, polymer materials need to be chosen with the objective of enhancing their resilience to weather and corrosion. Fluorocarbon coatings are a popular choice of polymer materials that offer robust weather resistance and anticorrosive properties and are commonly used to protect metal sculptures. For example, the iconic city sculpture *The Wind of May* (see Figure 8) in Qingdao, China, created by the renowned Chinese sculptor Huang Zhen using steel, stands 30 m tall, has a diameter of 27 m, and weighs over 500 tons. To protect the sculpture, it is coated with a fluorocarbon coating. Qingdao is a coastal city. The sculpture is located by the sea and is subject to long-term exposure to a humid, sunlit, and windy environment. To ensure that the steel sculpture is protected from corrosion, fluorocarbon coatings, which consist of fluorinated resin such as fluoroolefin polymer or fluorinated olefin copolymers and other monomers as the film-forming material, are utilized. These coatings are a modified form of fluororesin, contain a high number of stable molecular structures, and possess F-C bonds with a high bond energy of up to 486 kJ/mol. This makes them resistant to damage from heat, light, and physical or chemical factors and provides them with excellent weather resistance, corrosion resistance, stain resistance, heat resistance, chemical resistance, water and oil repellency, insulation, and low friction coefficients [67]. Due to these exceptional properties, fluorocarbon coatings are applied to the metal surface of the sculpture, where they adhere in a film-like manner, providing protection against environmental damage. 

When protecting sculptures made from materials that have low strength, fragile surfaces and are prone to damage, the primary consideration is to use high-performance polymer materials that can increase their surface strength. One commonly used polymer material for this purpose is polyurethane, which is an elastomeric material produced by the reaction of isocyanate and amino compounds. Polyurethane is known for its anti-corrosion, waterproofing, and abrasion resistance properties. By spraying polyurethane on the surface of sculptures made of soft materials, the sculpture’s surface texture can gain good flexibility and the surface strength of the sculpture is enhanced, protecting it from external impact and damage.

## 5. The Value and Shortcomings of Polymer Materials Applied to Contemporary Sculpture Art Creation

### 5.1. The Value of Polymer Materials Applied to the Creation of Contemporary Sculpture Art

In shaping, decorating, and protecting sculptural artworks, polymer materials have been widely involved in creating contemporary sculptural art, which is of great value and significance to the development of contemporary sculptural art.

First of all, the application of polymer materials in contemporary sculpture art creation has allowed artists to explore a diverse range of materials and expand their artistic vocabulary. This has led to further enrichment of the sculptural artists’ creative materials and artistic language. 

Secondly, the application of polymer materials has fostered a closer relationship between technology and sculpture art. Art has historically developed in tandem with technological progress, and the advent of synthetic polymer materials as a product of technological advancement, along with 3D printing technology, has transformed contemporary sculpture art creation. This has facilitated a seamless integration of technology and sculpture art creation.

Finally, the use of polymer materials has ultimately led to a decrease in expenses associated with the production of contemporary sculpture art. Material and processing expenses are the primary cost drivers in this domain. The cost and labor time associated with these resources are comparatively high. Consequently, creators have been exploring ways to minimize these expenses and expedite the creation process. A related study found that the application of polymer materials has been shown to significantly reduce production costs and expedite the creation cycle compared to traditional sculpture materials, which is instrumental in reducing the overall cost of contemporary sculpture art creation [3].

### 5.2. Shortcomings of Polymer Materials Applied to Contemporary Sculpture Art Creation

Although polymer materials are widely used in contemporary sculpture creation and possess various values and meanings, there are also certain drawbacks associated with their use.

First, polymer materials lack some of the spiritual and cultural connotations that traditional sculpture materials possess. As a result of their affordability, many modern sculptors opt for synthetic polymer materials as a replacement for more traditional materials. However, synthetic polymer materials are a byproduct of modern technology and, as such, do not possess the same cultural value as their traditional counterparts. If contemporary sculptors use these materials in a careless manner, their works may lack a certain artistic expression, ultimately detracting from the overall effectiveness of the sculpture.

Secondly, polymer materials have a shorter lifespan and pose environmental challenges. Compared to traditional sculpture materials such as metal and stone, the molecular structure of polymer materials is not stable; especially when synthetic polymer materials are used, there will be problems such as rapid aging, hardly degradation, and difficult repair. These issues can curtail the longevity of sculptures and harm their ecological setting. Additionally, numerous synthetic polymer materials are hazardous and may pose a risk to human health if inappropriately employed. Therefore, forthcoming research should concentrate on enhancing the durability of polymer sculpting materials, addressing the negative impact caused by polymer materials on humans and the environment during the creative process, and identifying eco-friendly and innocuous polymer materials for contemporary sculpture creation.

Finally, the research related to the application of polymer materials in creating contemporary sculpture art needs to be enriched. Despite the widespread employment of such materials within the field, the relevant research in this area is notably lacking. Upon reviewing the existing literature, it becomes apparent that there are only a handful of studies dedicated to the application of fiberglass materials in sculpture creation. Given that polymer materials have become an integral component of contemporary sculptural practice, the insufficient research in this domain may impede a comprehensive comprehension of their use by artists in the field. Ultimately, this shortfall in knowledge may prove detrimental to the progress and advancement of contemporary sculpture as a whole.

## 6. Conclusions and Prospects

### 6.1. Conclusions

Contemporary artists are able to effectively convey their creative ideas and present them perfectly with the strong support provided by polymer materials. These materials, with their diverse types, rich colors, and excellent properties, have become a popular choice for sculptors in their art creation. This paper presents a systematic exploration of the application of polymer materials in contemporary sculptural art and draws the following conclusions through the study:

(1) There are three forms of application of polymer materials in the creation of contemporary sculpture art: first for creating contemporary sculpture artworks; second for decorating contemporary sculpture artworks; third for protecting contemporary sculpture artworks. The use of polymer materials in the production of sculpture artworks is accomplished through three primary methods. The first method involves using polymer materials as replication models, utilizing remanufacturing molding techniques. GFRP is the most commonly used material for this purpose. The second method involves utilizing polymer materials as 3D printing materials and creating sculpture works through printing molding techniques. Thermoplastic filamentous materials, photosensitive resins, and polymer powders are the most commonly used materials for this purpose. The third method involves utilizing polymer materials as component materials and creating sculpture works through construction molding techniques. Natural and synthetic polymer materials are the commonly used materials for this method.

(2) The way to apply polymer materials to decorate contemporary sculpture artworks is mainly to color or imitate the texture of sculpture works. Among these materials, acrylic and spray paints are frequently employed, both of which can be utilized to great effect in decorating the sculptures.

(3) The application of polymer materials to protect contemporary sculptural artworks is commonly achieved through the application of protective films via spray coating, with fluorocarbon coating being the most prevalent. Especially for public sculptures situated in open-air public areas, fluorocarbon-coating can provide excellent protection.

(4) Despite their potential to offer contemporary sculpture artists a range of creative possibilities in the creation, decoration, and protection of their works, polymer materials are limited in their ability to convey spiritual and cultural meanings. Moreover, they have a shorter lifespan when compared to traditional sculpture materials and can pose environmental risks. While polymer materials have the potential to bring technology and sculpture closer together and reduce the cost of creating contemporary sculpture art, research in this area continues to lag behind.

### 6.2. Research Limitations and Future Research

Although the study in this paper explores the application of polymer materials in the creation of contemporary sculptural art in a more systematic manner, there are inevitably some shortcomings, which also represent potential directions for future research.

(1) While the study explores various ways of using polymer materials to create, decorate, and protect contemporary sculpture art, it does not offer a detailed discussion of specific application processes. Future investigations could focus on the specific application processes of polymer materials in contemporary sculpture art to better assist creators. 

(2) Since the study primarily examines the application of polymer materials in the creation of contemporary sculpture art, but does not address issues such as the short lifespan and environmental impact of sculptures made with these materials. As the use of polymer materials in contemporary art increases, research in related fields should provide recommendations for future research.

(3) Although the study systematically examines the application of polymer materials in contemporary sculpture art, its research is relatively macroscopic. While there have been many successful cases of using polymer materials in contemporary sculpture art, relevant research has lagged behind. Thus, further in-depth research in related fields is necessary to fully explore the potential of combining polymer material science and contemporary sculpture art.

Technology is an important driving force for art. The development and application of polymer materials have brought about substantial implications for contemporary sculpture art. With the continuous development of polymer materials and 3D printing technology, the limitations of polymer materials in contemporary sculpture will be improved. Subsequently, research in this field is expected to become more extensive and comprehensive, providing contemporary sculpture artists with more profound insights and practical assistance.

## Figures and Tables

**Table 1:** Comparison of basic physical properties of commonly used metal materials and GFRP in sculpture creation [23,27,28,29,30].

Materials	Density	Tensile Strength	Elastic Modulus	Thermal Expansion Coefficient
GFRP	1.4~2.5 g/cm^3^	>150 Mpa	10~25 Gpa	2.7~7.2 (10^−6^/°C)
Copper	8.92~8.96 g/cm^3^	200~360 Mpa	90~130 Gpa	7.8~9.8 (10^−6^/°C)
Stainless Steel	7.75~7.93 g/cm^3^	>520 Mpa	190 Gpa	6.4~10.4 (10^−6^/°C)
Iron	7.83~7.87 g/cm^3^	220~260 Mpa	151~160 Gpa	11.6~12.1 (10^−6^/°C)

**Table 2:** Comparison of basic physical properties of commonly used metal materials in GFRP and sculpture creation [38,39,40,41].

Materials	Viscosity 25 °C	Shrinkage 25 °C	Acid Value	Solid Content
191# Unsaturated resin	0.25~0.45 Pa·s	1.6 %	16~36 mg KOH/g	67~75%
196# Unsaturated resin	0.65~1.15 Pa·s	2.1%	17~25 mg KOH/g	60~70%
Epoxy resin	0.60~0.95 Pa·s	1.8%	30~40 mg KOH/g	68~72%

**Table 3:** Comparison of several polymer materials commonly used for 3D printing sculpture art [42,43,44,47].

Materials	Material Properties	Applicable Technologies	Printing Results
ABS	Good thermal melting performance, easy extrusion, high impact resistance, high heat resistance, strong low-temperature resistance, and good corrosion resistance.	FDM	Stable size and quality, good glossiness, toughness, and high strength.
PLA	High strength, good thermal plasticity, fiber-forming ability, high transparency, low degradability, no odor, and low shrinkage rate.	FDM	Stable size and quality, smooth surface, but high brittleness.
PVA	Good toughness, thermal stability, water solubility, good transparency, low fluidity, and easy processing.	FDM	High glossiness and whiteness, high hardness, and easy to clean.
PC	High strength, low shrinkage rate, good impact resistance, toughness, optical properties, abrasion resistance, oxidation resistance, and stain resistance.	FDM	High transparency and stability, solid and durable quality, but low surface precision.
Photosensitive resin	Low shrinkage, good flexibility, low mechanical strength, heat resistance and weather resistance, can be used to print complex structures.	SLA	High surface precision, good detail performance, and high stability and quality.
Thermoplastic polymer powder	High tensile strength, impact strength, flexural strength and modulus of mixed powder products, low water absorption, easy to process.	SLS	Good appearance quality and high fineness.
Thermoset polymer powder	It has the same characteristics as thermosetting resin, such as high strength and fire resistance.	SLS	High stability, smooth surface, light quality, and more delicate.
Polymer composite powder	High tensile strength, bending strength and modulus of the mixed powder, good mechanical properties, good fluidity.	SLS	Higher sintering rate and high dimensional accuracy.

**Table 4:** Comparison of two types of polymer binders commonly used in sculpture art creation [42,43,44].

Materials	Preparation Method	Properties	Applicable Scope
Epoxy resin AB adhesive	It is made by combining two or more epoxy resins (Group A) with a curing agent such as fatty amine.	Can be cured at low or room temperature, with a fast curing speed; the shear strength after curing is ≥18 Mpa; the working temperature after curing is -50~+180 °C.	Suitable for gluing high temperature resistant metals, ceramics, and other materials.
EVA resin hot-melt adhesive	The resin is formed by the copolymerization of ethylene and vinyl acetate under high pressure, blended with thickening agents, viscosity regulators, antioxidants, etc.	Reusable; fast curing speed; viscosity (room temperature 25 °C): 80~100 Pa.s; extremely resistant to low temperatures (−70 °C).	Suitable for bonding EVA material, wood, leather, EPE, and other materials.

## Data Availability

All data supporting this study have been listed in the article.
